# Particulate matter exposure and health impacts of urban cyclists: a randomized crossover study

**DOI:** 10.1186/s12940-018-0424-8

**Published:** 2018-11-14

**Authors:** Christie A. Cole, Christopher Carlsten, Michael Koehle, Michael Brauer

**Affiliations:** 1 School of Population and Public Health, University of British Columbia, 2206 East Mall, Vancouver, BC V6T 1Z3 Canada; 2 Air Pollution Exposure Lab, Department of Medicine, University of British Columbia, 2775 Laurel Street 7th Floor, Vancouver, BC V5Z 1M9 Canada; 3 School of Kinesiology and Division of Sport & Exercise Medicine, University of British Columbia, 2176 Health Sciences Mall, Vancouver, BC V6T 1Z3 Canada

**Keywords:** Cycling, Endothelial function, Oxidative stress, Inflammation, Lung function, Air pollution, Particulate matter

## Abstract

**Background:**

Cycling and other forms of active transportation provide health benefits via increased physical activity. However, direct evidence of the extent to which these benefits may be offset by exposure and intake of traffic-related air pollution is limited. The purpose of this study is to measure changes in endothelial function, measures of oxidative stress and inflammation, and lung function in healthy participants before and after cycling along a high- and low- traffic route.

**Methods:**

Participants (*n* = 38) bicycled for 1 h along a Downtown and a Residential designated bicycle route in a randomized crossover trial. Heart rate, power output, particulate matter air pollution (PM_10_, PM_2.5_, and PM_1_) and particle number concentration (PNC) were measured. Lung function, endothelial function (reactive hyperemia index, RHI), C-reactive protein, interleukin-6, and 8-hydroxy-2′-deoxyguanosine were assessed within one hour pre- and post-trial.

**Results:**

Geometric mean PNC exposures and intakes were higher along the Downtown (exposure = 16,226 particles/cm^3^; intake = 4.54 × 10^10^ particles) compared to the Residential route (exposure = 9367 particles/cm^3^; intake = 3.13 × 10^10^ particles). RHI decreased following cycling along the Downtown route and increased on the Residential route; in mixed linear regression models, the (post-pre) change in RHI was 21% lower following cycling on the Downtown versus the Residential route (−0.43, 95% CI: -0.79, −0.079) but RHI decreases were not associated with measured exposure or intake of air pollutants. The differences in RHI by route were larger amongst females and older participants. No consistent associations were observed for any of the other outcome measures.

**Conclusions:**

Although PNC exposures and intakes were higher along the Downtown route, the lack of association between air pollutant exposure or intake with RHI and other measures suggests other exposures related to cycling on the Downtown route may have been influential in the observed differences between routes in RHI.

**Trial registration:**

ClinicalTrials.gov, NCT01708356. Registered 16 October 2012.

## Background

The World Health Organization (WHO) identifies sedentary lifestyles as a major risk factor for global mortality and chronic disease [1]. Only 15% of Canadian adults [2] meet WHO and Canadian Physical Activity Guidelines of 150 min of moderate-to-vigorous physical activity per week in bouts of 10 min or more [1, 3]. Cycling is a form of active transportation that it is relatively accessible to all socio-economic classes and addresses typical utilitarian transportation distances of < 8 km [4, 5]. In addition to its potential as a strategy to reduce physical inactivity [6], cycling provides further societal benefit as a form of transportation that does not produce harmful emissions [7–10].

Despite the potential benefits of cycling, there are concerns regarding adverse health impacts to cyclists due to increased inhalation of traffic-related air pollution especially in urban areas where cyclists often travel in close proximity to road traffic [11–13]. Studies have consistently shown the physiological impacts of acute exposure to particulate matter (PM), linking it with changes in vascular tone [14–16], cardiovascular morbidity and mortality [17, 18], oxidative stress [19], pulmonary inflammation [20], and stimulation of pulmonary irritant receptors [21, 22]. However, these studies only serve to demonstrate the acute health effects of PM exposure in the home and laboratory, environments that lack elements present during a cycling trip that may further impact the effect of PM exposure on health. For instance, increased respiration rates due to physical effort involved while cycling leads to increased inhalation of polluted air [23, 24].

While several studies have documented unfavorable physiological changes, such as to lung function and heart rate variability [25–27], as well as increases in inflammatory biomarkers [28, 29] amongst cyclists exposed to air pollution, few such studies have attempted to quantify exposure and intake levels of air pollutants and the resulting effect on acute health measurements. There is also limited understanding of the health impacts due to variations in pollution concentrations along cycling routes. Therefore, this study aims to assess the acute health impacts of cycling along a low versus a high traffic route using a randomized crossover design. This study compares exposure to, and intake of, PM while cycling, using a number of biochemical and clinical parameters measured before and after traveling along two routes. Specific measurements were selected to represent a set of well validated biochemical markers of inflammation (interleukin-6 [IL-6] and C-reactive protein [CRP]) and oxidative stress (8-hydroxy-2′-deoxyguanosine [8-OHdG]), along with clinical vascular (reactive hyperemia by EndoPAT) and pulmonary function measures.

## Methods

### Participants and study design

Healthy adult (ages 19–39) participants were recruited using advertisements posted to local cycling and university bulletin boards and along cycling routes in the city of Vancouver, Canada. Participants were eligible if they were non-smokers, not diagnosed with or taking a medication for any respiratory or cardiovascular condition, not exposed to environmental tobacco smoke in the home or otherwise exposed to significant respiratory exposures in the workplace. Participants with seasonal allergies were asked to participate at a time of the year when they were asymptomatic. Females were tested during days 1–8 of their follicular phase, while those using monocyclic oral contraceptives were tested on days where they took an active pill to ensure consistent hormonal status. Written informed consent was obtained for each participant after a session given to familiarize each person with the equipment and protocols, prior to commencing the first cycling trial. The Health Canada (certificate #2011–0009) and University of British Columbia Clinical Research (#H10–00902) Ethics Boards approved this study. The study was registered with ClinicalTrials.gov (NCT01708356).

All cycling trials began and ended at the Vancouver General Hospital in central Vancouver, British Columbia during May– November 2010 and May– November 2011. Metro Vancouver has an annual mean particle number concentration of 18,200 particles/cm^3^ (pt/cm³) (standard deviation = 15,900 pt/cm^3^) (from 2009 and 2010 data) [30], an annual mean PM_2.5_ concentration of 4.3 μg/m^3^ at the T2:Vancouver- Kitsilano station, and an annual mean PM_10_ concentration of 10.5 μg/m^3^ at the nearest monitoring location (T24: Burnaby North ) [31]. Trials were conducted between 700 and 1600 h, and each participant completed both trials at the same time of day (+/− 1 h), scheduled 2 to 6 weeks apart. Two routes were selected using a recent study that obtained particulate matter exposure measurements from designated bicycle routes in Vancouver [13], with consideration of nearby land-use categories in order to select one predominantly residential use (“Residential”) route and one predominantly commercial or higher density residential (“Downtown”) route. The mid-point of the Downtown ride (Dunsmuir Street at Richards Street, 2011 through-traffic counts) had traffic counts between 7.8 to 9.9 times that of the traffic on a typical section (Ontario Street at 36th Street, based on 2006 traffic counts) along the Residential Route. Route order was assigned randomly. The Downtown route was a 9.7 km loop, and was always traveled in a counter-clockwise direction, with a section of protected bike lane that could be repeated if the cyclist was a faster rider. Most participants repeated the protected bike lane section, resulting in a total elevation gain of 127 m over three major uphill segments (with the most significant segments being 29 m, 26 m, and 26 m in elevation gain) [32]. The Residential route was a 12.0 km loop that was traveled in a clockwise direction for some trials (120 m of elevation gain, from 2 major uphill segments of 49 m and 26 m), or as an out-and-back ride in a counter-clockwise direction, to the far south-east corner of the route before reversing direction (172 m of elevation gain, over 3 major uphill segments of 41 m, 36 m, and 18 m) [32]. A trailing research assistant (also on bike) provided wayfinding and timing directions with the aim of facilitating a 60-min ride, rather than completing a specific distance. Questionnaires prior to the beginning of each trial were used to screen for allergy or cold symptoms, to confirm that each participant had limited alcohol and caffeine consumption, and to confirm that each participant consumed the same meals prior to the trials. Participants were also asked to travel to the study site using the same method of transportation on both trial days.

### Exercise and exposure monitoring

Two bicycles of the same model (KHS, Flite 250, Los Angeles, USA), one each of small and large frame size, were used in all trials. A PowerTap Comp (PowerTap, Madison, WI, USA) wiring set which automatically recorded at 3-s intervals during the ride, was installed on the handlebars of each bicycle to measure power output, cadence, and heart rate from a chest-worn heart rate strap. A single PowerTap hub was installed on a wheel that could be transferred between the two bicycles. A P-trak (Ultrafine Particle Counter 8525, TSI Inc., Shoreview, MN, USA), with the *tilt* sensor removed was mounted into a wire pannier (Swagman Fat Folding Basket, Swagman Racks, Penticton, BC, CAN) by placing a horizontal bar through the handle of the monitor and stabilized using elastic cords and metal bearings, thus allowing the P-trak to remain horizontal despite changing inclines. The P-trak recorded particle number (0.02–1 μm) concentration (PNC) at one-second intervals. A GRIMM Dust Monitor (Model 1.108, GRIMM Technologies, Inc. Douglasville, Georgia, USA) placed into a separate rear pannier measured PM_10_, PM_2.5,_ and PM_1_ concentrations at 6-s intervals. Sampling inlets for both monitors were secured along the top tube of the bicycle, extending to the centre of the handlebars. A GPS logger (DG-100 GPS DataLogger, GlobalSat WorldCom Corporation, New Taipei City, Taiwan) recorded the location of the bicycle at 6-s intervals. To align measurements from all instruments, data collected at intervals longer than 1 s was applied to the closest one second time point until a new data point was available, carried for a maximum of 6 s.

### Physiological measurements

All measurements were completed within one hour prior to the beginning of each bicycle ride, and were repeated approximately 15 min after the cyclists’ return and completed within 90 min of ride termination. Tests were administered in uniform order (endothelial function, followed by spirometry, followed by bloodwork) before and after each ride, with exceptions noted and replicated for a given subject when possible. For a given individual, tests for the second trial were performed within one hour of the same time of day as they were performed in the first trial. Endothelial function was measured using the EndoPAT 2000 device (Itamar Medical Ltd., Caesarea, Israel) to measure RHI, following the five-minute occlusion procedure recommended in the user manual. Lung function was measured with a KoKo spirometer (nSpire Health, Longmont, Colorado, USA), following American Thoracic Society standards [33]. Five milliliters of blood were collected, centrifuged, and frozen prior to analysis for CRP (Dimension Vista® System Flex® reagent cartridge for high sensitivity CRP, Siemens Healthcare Diagnostics Products GmbH 2009), IL-6 (Quantikine® ELISA Human IL-6 Immunoassay D6050, R&D Systems, Inc. Minneapolis, MN, USA) and 8-OHdG (Highly Sensitive 8-OHdG Check ELISA method, Japan Institute for the Control of Aging, Nikken Seil Co. Ltd., Fukuroi, Shizuoka, Japan). Increased RHI indicates improved endothelial function and increases in spirometric measures indicate improved lung function, whereas increases in any of the blood measures indicate increased oxidative stress and/or inflammation.

The first 15 participants completed an abbreviated indoor cycling test after the one-hour ride. Minute ventilation was recorded while cycling indoors at the mean heart rate recorded during the ride along each outdoor route. All indoor cycling tests were completed on an adjustable Velotron Dynafit Pro cycle ergometer (Racermate Inc., Seattle WA). The remaining 23 participants completed a step-wise submaximal exercise test, in increments of 20 watts every two minutes for females and 30 watts every two minutes for males. Heart rate using a Polar HR sensor strap (Polar s810i, Polar Electro, Finland), and minute ventilation (${\dot{\mathrm{V}}}_{\mathrm{E}}$) using a respirometer (Spirolab II, Medical International Research, Rome, Italy) were recorded during the second minute at every resistance level throughout the heart rate range experienced by each participant. Heart rate data from each participant’s submaximal test results were used to estimate a subject-specific HR-${\dot{\mathrm{V}}}_{\mathrm{E}}$ relationship, which was then used to estimate instantaneous and mean ${\dot{\mathrm{V}}}_{\mathrm{E}}$ for each outdoor trial for each participant; ${\dot{\mathrm{V}}}_{\mathrm{E}}$ was estimated at each data point during outdoor cycling trials to calculate the total volume of inhaled air, and therefore PM intake values.

### Statistical analysis

Only participants who completed trials on both routes were included in the data analysis. Out of 76 exposure trials, 6 trials had missing exposure data. Health measures were analyzed in the group results only when complete pairs of pre and post measurements were available; incomplete pairs were excluded. Data were analyzed by paired t-tests comparing the two trials for each participant, and in mixed effects regression models. Fixed variables (e.g. bivariate variables comparing the Residential and Downtown routes, or continuous variables such as air pollution exposure or intake values), and random variables (participants) were modeled in R [34] using the lme4 package [35] to predict changes in clinical measures. Both exposure and pollutant intake were considered in analyses.

Intake was estimated for each participant by summing the product of each 1-s pollutant concentration (in pt/cm^3^ or μg/m^3^) by the volume of air inhaled each second derived from the heart rate data (the mean ${\dot{\mathrm{V}}}_{\mathrm{E}}$ of the entire trial, converted to L/s). As high correlations were measured between the PM_10_, PM_2.5_, and PM_1_ measurements, (Pearson product-moment correlation coefficients of 0.96 to 0.99) only results for the models that include PM_2.5_ and PNC concentrations are presented. Pollutant concentration distributions within each ride were right-skewed and summarized by the geometric mean (GM). Four mixed-effects models (with participant included as a random effect) were built in order to evaluate the effect of route differences (model 1), the effects of pollutant exposure (model 2) or intake (model 3) and the effect of pollutant intake while including route in the same model (model 4):

1. Change in clinical measurement = ß _Route_ + participant.
2. Change in clinical measurement = ß _GM exposure_ + participant.
3. Change in clinical measurement = ß _Pollutant intake_ + participant.
4. Change in clinical measurement = ß _Route_ + ß _Pollutant intake_ + participant

Effect modification was explored by modeling body mass index (BMI: lower and upper half, stratified at the median), age (younger half and older half, stratified at the median), and sex (female or male) variables with the Route variable scaled to the Residential route.

## Results

Mean age of the participants (*n* = 28 male, 10 female) was 29 ± 6 years (range 20–39 years), and mean BMI was 22.8 ± 2.0 kg/m^2^ (Table 1). Most participants (87%) used the same method of transportation to arrive at the study site on both testing days. Three participants reported taking prescribed thyroid or anxiety medications before completing each of their trials.

**Table 1:** Descriptive data of participants and summary of physiological baseline measurements

Variable	Baseline mean [SD] or total	Range
Total participants (male, female)	38 (28, 10)	–
Age (years)	Overall 29 [5.6]; median = 29 Male mean = 29 Female mean = 31	20–39
BMI (kg/m^2^)	22.8 [2.0]; median = 22.8	18.3–28.0
Systolic BP (mmHg)	117 [9]	90–139
Diastolic BP (mmHg)	67 [6]	52–83
Reactive Hyperemia Index (RHI)	2.02 [0.64]	1.29–4.28
CRP (mg/dL)	0.85 [1.2]	0.11–7.7
IL-6 (pg/mL)	3.6 [4.3]	0.023–16
8-OHdG (ng/mL)	0.20 [0.12]	0.012–0.73
# participants whose first trial was along the Downtown route	17	–
Morning test (session end by 12:30 pm)	23	–
Cold Questionnaire score (≥ 3 probable viral infections)	0.4 [0.9]	0–3

Overall mean ride time for all trials along both routes was 63.9 and 62.9 min on the Downtown and Residential routes, respectively (range = 56–73 min). Mean concentrations of PNC were significantly higher along the Downtown route (16,870 pt/cm³) compared to the Residential route (10,840 pt/cm³, Table 2). Higher concentrations of other particle measures were also observed in trials along the Downtown compared to the Residential route (Table 2).

**Table 2:** Air pollution exposure measurements, as calculated from means of each trial

Pollutant^a^	Downtown route			Residential route			Ratio of downtown: residential route^b^	95% CI of the downtown – residential difference
	Mean [SD]	Median; range (min, max)	GM [GSD]	Mean [SD]	Median; range (min, max)	GM [GSD]		
PNC (pt/cm³)	16,870 [4838]	15,740 (9597, 29,060)	16,226 [1.33]	10,840 [5159]	10,280 (977, 22,130)	9367 [1.86]	1.53	3695, 8376
PM_1_ (μg/m^3^)	5.0 [4.2]	3.7 (1.2, 20)	3.8 [2.0]	3.8 [2.9]	2.9 (0.48, 12)	2.9 [2.1]	1.3	−0.44, 2.9
PM_2.5_ (μg/m^3^)	7.3 [5.3]	5.5 (2.4, 24)	6.0 [1.9]	5.8 [3.7]	4.4 (1.1, 15)	4.7 [1.9]	1.3	−0.58, 3.6
PM_10_ (μg/m^3^)	13 [7.3]	11 (4.3, 33)	11 [1.8]	9.9 [5.6]	8.8 (2.2, 29)	8.4 [1.8]	1.2	−0.14, 5.9

^a^Complete PM data was missing for 6 trials with PNC data missing for 3 trials and PM data missing for 3 trials

^b^Ratio of the median of the Downtown Route compared to the median of the Residential Route

Male participants experienced mean ${\dot{\mathrm{V}}}_{\mathrm{E}}$ of 47.9 (SD = 15.0) L/min during all trials, while female participants experienced mean ${\dot{\mathrm{V}}}_{\mathrm{E}}$ of 40.4 (SD = 11.0) L/min. Measurements from a subset of 22 cyclists permitted the comparison of estimated ${\dot{\mathrm{V}}}_{\mathrm{E}}$ ratio during a cycling trial compared to at rest (Table 3). The overall mean ${\dot{\mathrm{V}}}_{\mathrm{E}}$ ratio (cycling: rest) for the group was 3.5. Cyclists had a higher ${\dot{\mathrm{V}}}_{\mathrm{E}}$ ratio (3.8) when cycling along the Residential route compared to the Downtown route (3.3). The mean volume of air estimated to be inhaled by participants during a single cycling trial was 2900 L, and ranged from 820 to 4700 L.

**Table 3:** Air intake parameters by sex and by route. Ventilation averages with pollution concentration at each time point determined estimated values

Participant group mean or route mean	Ride time (minutes) [sd]	Mean ${\dot{\mathrm{V}}}_{\mathrm{E}}$ in L/min at rest [sd]	Mean ${\dot{\mathrm{V}}}_{\mathrm{E}}$ in L/min during ride by HR [sd]	Estimated intake of PNC during trial (particles) [sd]	Estimated volume of air inhaled at rest during equivalent ride time (Litres) (resting ${\dot{\mathrm{V}}}_{\mathrm{E}}$ x ride time) [sd]	Estimated volume of air inhaled during ride (Litres) (ride mean ${\dot{\mathrm{V}}}_{\mathrm{E}}$ x ride time) [sd]	Ratio of ${\dot{\mathrm{V}}}_{\mathrm{E}}$ riding compared to at rest [sd]
Males (*n* = 28)	63.6 [2.69]	15.8 [5.16]^a^	47.9 [15.0]	3.86 × 10^10^ [1.72 × 10^10^]	1107 [317]	3000 [961]	3.5 [2.8]^a^
Females (*n* = 10)	63.1 [3.35]	10.4 [1.76]^a^	40.4 [10.9]	3.86 × 10^10^ [2.21 × 10^10^]	827 [157]	2540 [946]	3.4 [1.1]^a^
Downtown	63.9 [3.42]	–	44.8 [14.0]	4.54 × 10^10^ [1.79 × 10^10^]	–	2840 [688]	3.3 [2.2]^a^
Residential	62.9 [3.57]	–	47.8 [14.3]	3.13 × 10^10^ [1.61 × 10^10^]	–	3020 [859]	3.8 [2.9]^a^
Overall	63.4	14.6 [5.13]^a^	46.1 [14.5]	3.86 × 10^10^ [1.95 × 10^10^]	1046	2890 [922]	3.5 [3.5]^a^

^a^Means calculated using complete pairs only; ${\dot{\mathrm{V}}}_{\mathrm{E}}$ in L/min at rest was only available for 22 participants (17 males, 5 females)

Power output was lower along the Downtown route compared to the Residential route (mean difference = 9.9 watts, 95% CI: 18, 1.8 watts). Mean heart rate was also slightly lower on the Downtown route (mean difference = 4.5 beats per minute, 95% CI: 8.6, 0.3 beats per minute). These differences could be attributed to the higher number of stops and intersections along the Downtown route.

**Fig. 1:**
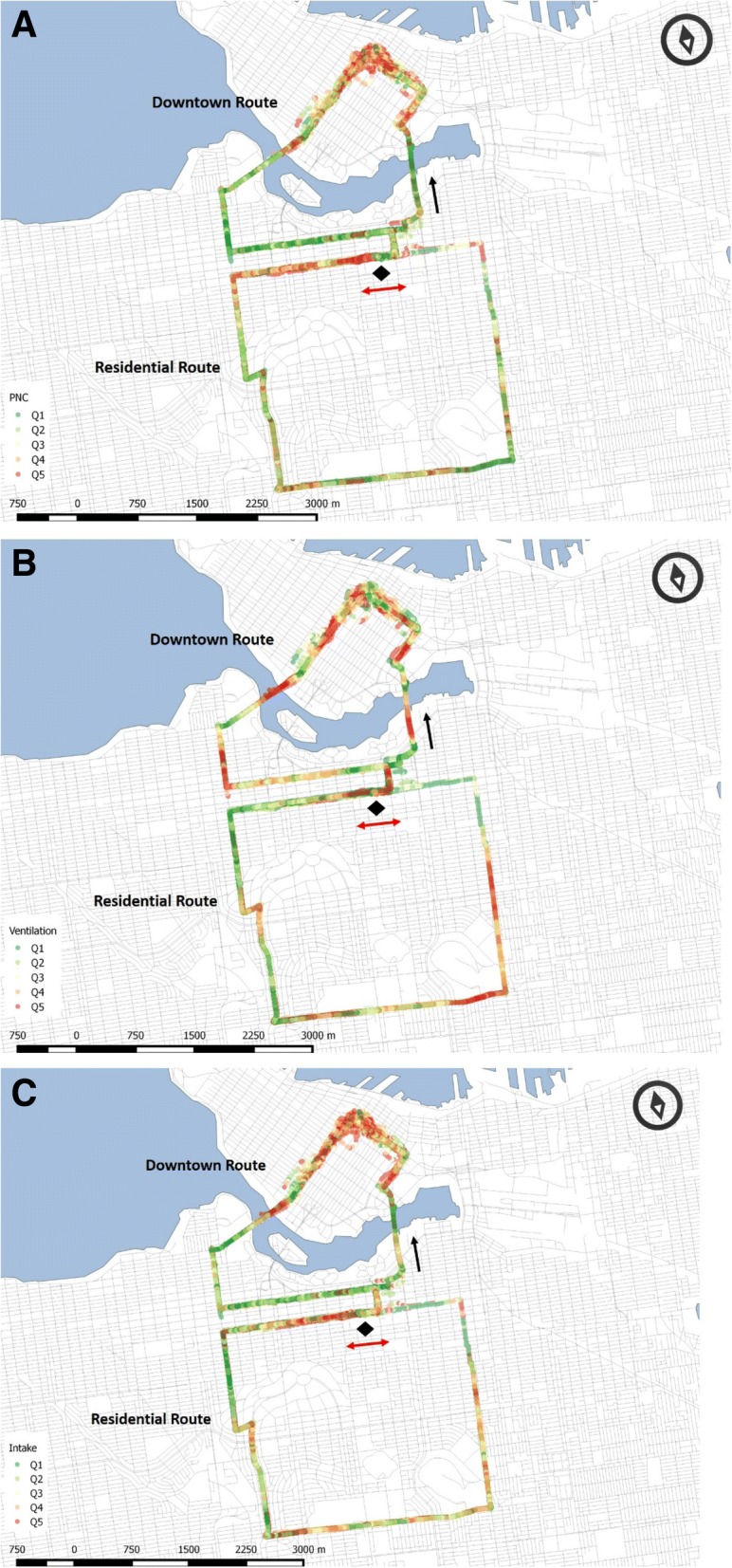
Quintiles for each individual cycling trial (of the 22 participants with complete heart rate and minute ventilation profiles), normalized across all participants, of the locations of highest and lowest **a** PNC levels, **b** Ventilation, **c** Intake (PNC x Ventilation). The start location is indicated by a diamond and arrows indicate the direction of travel (the Residential route was travelled in both directions)

Figure 1 displays the relationship between the spatial patterns in exposure (Fig. 1a), ventilation (Fig. 1b) and intake (Fig. 1c) for the 22 participants (as quintiles of each variable within each trial, normalized across all participants) with complete heart rate and minute ventilation profiles. The highest exposures occur at the northwest corner of the Downtown route and along the northern portion of the Residential route. For ventilation, however, the highest levels occur when cycling uphill, in a number of locations (for example, when cycling over the westernmost bridge leading away from the central business district and in the southeast segment of the Residential route). Intake values are highest, as expected, in locations with both high PNC and high ventilation, for example at the northern section of the westernmost bridge and in the middle of the northern segment of the Residential route. Given that the overall mean ${\dot{\mathrm{V}}}_{\mathrm{E}}$ ratio (cycling: rest) for the group was 3.5 and can therefore lead to 3.5-fold variation in intake for the same level of exposure, these results illustrate the importance of considering both ${\dot{\mathrm{V}}}_{\mathrm{E}}$ and exposure.

Paired t-tests indicated no changes in lung function after cycling along either route, with the exception of the forced expiratory flow (FEF_25__-__75_) measurement, which increased after cycling along the Downtown route.

Endothelial function increased by mean RHI of 0.25 after cycling along the Residential route, and decreased by mean RHI of 0.18 after cycling along the Downtown route. There was a difference in the change (post - pre) in RHI between the Residential compared to the Downtown route (−0.39, 95% CI: -0.77, −0.017, Table 4), suggesting a route-dependent impact on the level of improvement in endothelial function following cycling. Minor changes to blood biomarkers were observed when comparing post-pre measures along both routes, with small net increases in IL-6 and 8-OHdG after cycling the Downtown route compared to the Residential route (Table 4).

**Table 4:** Clinical measurement summary by route (Downtown and Residential) of post- and pre- cycling clinical measurements

Variable	Downtown route	Residential route	∆ Downtown - ∆ Residential
	Change (post-pre) Mean [∆ Downtown] (95% CI)	Change (post-pre) Mean [∆ Residential] (95% CI)	Mean difference (95% CI)
Endothelial Function- EndoPAT™			
RHI	−0.18 (−0.46, 0.11)	0.25 (0.03, 0.47)	−0.39 (−0.77, −0.017)
Spirometry (unit)			
FVC (mL)	46 (−3.8, 97)	21 (−84, 130)	28 (−77, 134)
FEV_1_ (mL)	46 (6.6, 86)	49 (−11, 110)	−0.81 (−62, 64)
FEF_25–75_ (mL/s)	110 (23, 190)	82 (−59, 220)	0.024 (−0.13, 0.18)
Blood Measures (unit)			
CRP (μg/dL)	8.8 (−12, 29)	6.6 (−30, 43)	2.4 (−44, 49)
IL-6 (pg/ml)	0.55 (−0.59, 1.68)	−0.61 (−1.8, 0.57)	1.13 (−0.82, 3.1)
8-OHdG (ng/ml)	−0.00045 (−0.040, 0.040)	−0.031 (−0.071, 0.0082)	0.029 (−0.023, 0.081)

Paired spirometry data was missing for 2 participants and paired endothelial function data missing for 4

There were some indications of small increases in IL-6 associated with exposure to PNC and PM_2.5_ while cycling (Table 5), although confidence intervals crossed zero. Similarly, there were indications of associations between PNC exposure and a small decrease in FVC (Table 5). Exposure to PM_2.5_ appeared to contribute to a reduction in FEV_1_, but was not seen in association with PNC exposure (Table 5). RHI decreases were associated with cycling along the downtown route in models where only route was considered (Table 6, model 1) as well as in models including pollutant intake (Table 6, model 4), whereas estimates for pollutant intake showed no evidence of association with RHI (Table 6, model 3 and model 4). This suggests that the observed associations between route and RHI were independent of pollutant exposure or intake. The decrease of 0.43 units for cycling on the Downtown route was equivalent to a 21% decrease (based on mean pre-cycling RHI of 2.0 across all participants) in RHI. Other endpoints showed no associations with pollutant intake or route (Table 6).

**Table 5:** Mixed effects (model 2) coefficients of subclinical health measure, modeled using the GM concentration of PNC or PM_2.5_ exposures for each trial

Outcome measurement	GM of PNC ß-coefficient	95% CI	GM of PM_2.5_ ß-coefficient	95% CI
RHI	0.066	−0.22, 0.35	−0.051	−0.25, 0.15
FEV_1_ (mL)	−4.1	−53, 45	−32	−66, 3.0
FVC (mL)	−63	− 145, 19	−41	−102, 19
CRP (μg/dL)	8.7	−14, 32	2.2	−14, 19
IL-6 (pg/mL)	0.78	−0.45, 2.0	0.64	−0.20, 1.5
8-OHdG (ng/mL)	0.027	−0.013, 0.067	0.010	−0.019, 0.039

ß-coefficient values are presented for an interquartile range change in PNC (7637 pt/cm³) or PM_2.5_ (4.7 μg/m^3^) exposure

**Table 6:** Effects estimates per IQR change in PNC intake, PM_2.5_ intake and route (Downtown, with Residential as the reference) modeled for clinical measures

Clinical measure	[Model 3] PNC intake ß-coefficient (95% CI)	[Model 4] PNC intake ß-coefficient in model adjusting for route (95% CI)	Downtown route ß-coefficient (95% CI)	[Model 3] PM_2.5_ intake ß-coefficient (95% CI)	[Model 4] PM_2.5_ intake ß-coefficient in model adjusting for route (95% CI)	Downtown route ß-coefficient (95% CI)	[Model 1] Downtown route ß-coefficient (95% CI)
RHI	0.050 (−0.20, 0.30)	0.15 (−0.10, 0.40)	−0.50 (−0.90, −0.10)	0.0010 (−0.091, 0.093)	0.017 (−0.073, 0.11)	−0.46 (−0.83, −0.089)	−0.43 (−0.79, −0.079)
FEV_1_ (mL)	20 (−26, 66)	21 (−29, 70)	−3.3 (−74, 67)	−0.64 (−17, 16)	−0.20 (−17, 16)	−13 (−80, 55)	0.81 (−60, 62)
FVC (mL)	−33 (− 109, 43)	−46 (−130, 34)	55 (−60, 170)	−0.79 (−29, 27)	−1.5 (−30, 27)	20 (−91, 130)	28 (−74, 130)
CRP (μg/dL)	1.7 (−26, 30)	1.2 (−29, 31)	2.1 (−44, 49)	−0.072 (−7.7, 7.6)	−0.77 (−8.5, 7.0)	19 (−13, 50)	2.3 (−39, 43)
IL-6 (pg/mL)	0.55 (−0.53, 1.6)	0.36 (−0.76, 1.5)	1.1 (−0.62, 2.8)	0.17 (−0.22, 0.57)	0.13 (−0.26, 0.53)	1.2 (−0.52, 2.8)	1.2 (−0.43, 2.7)
8-OHdG (ng/mL)	15 (−25, 54)	2.8 (−41, 47)	32 (−25, 88)	2.2 (−11, 15)	−0.037 (−13, 13)	39 (−12, 90)	30 (−17, 78)

ß-coefficient values are presented for an interquartile range change in PNC (2.5 × 10^10^ particles) or PM_2.5_ (15.0 μg) intake

**Fig. 2:**
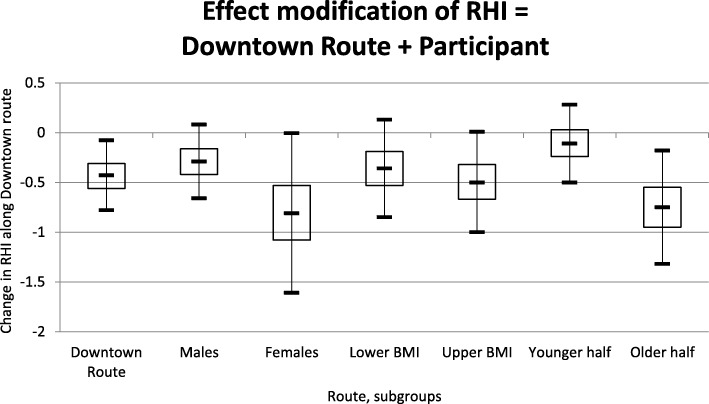
Effect modification of RHI by variables including sex, BMI, and age. BMI and age were stratified by those above and below the median level (BMI: 22.8 kg/m^2^, age: 29 years)

Effect modification for RHI change by route (model 1) was analyzed by sex and age, dichotomized at the median (≤ 28 versus > 29 years), shown in Fig. 2. The effect of route on RHI change was larger amongst females (ß = −0.81, 95% CI: -1.6, −0.0048) compared to males (ß = −0.29, 95% CI: -0.66, 0.081). Similarly, the effect of route was larger amongst the older half of the participants (ß = −0.75, 95% CI: -1.3, −0.18) compared to younger half of the participants (ß = −0.11, 95% CI: -0.50, 0.28). There was no evidence of effect modification by BMI.

## Discussion

This study compared exposure and intake levels of air pollutants while cycling along low versus high traffic routes, and measured acute health impacts of cycling along these routes. Mean exposure and intake levels of air pollutants were observed to be higher along the high traffic (Downtown) route. Improvements in endothelial function were observed after cycling along the low traffic Residential route, while cycling along the Downtown route led to a mean decrease in endothelial function measures. These decreases were larger amongst females and older participants. No consistent changes in lung function or in blood biomarkers of systemic inflammation and oxidative stress were observed after cycling either route in this study.

The lack of agreement between measures of endothelial function and blood biomarkers of oxidative stress and inflammation in this study, despite the higher measured levels of air pollutants along the high traffic route, suggests that there are other factors besides air pollution which may have affected endothelial function. Few studies have evaluated the effects of air pollution on endothelial function and blood biomarkers concurrently, and these few studies have reported conflicting results on the effect of air pollutant exposure on endothelial function [36–41]. Consistent with the findings of this study, most of these studies found non-significant changes to the biomarkers of CRP [36, 42], IL-6 [28, 29, 36, 41, 42], and 8-OHdG [43] at different levels of PM exposure.

It is possible that the observed improvement in endothelial function when cycling the Residential route was due to higher physical activity intensity as indicated by the higher measured power output for this route. Cyclists encountered more frequent stopping conditions (e.g. traffic lights, intersections) along the Downtown route, leading to lower mean workloads and opportunities for heart rates to decrease during these intermittent rest periods. The total ride time was nearly the same for both routes, differing by only one minute, for an overall mean ride time of 63.4 min.

Other potential influencing factors may include the differences in noise levels and cyclist stress or anxiety levels along these two routes. Noise has been shown to impact measures of cardiovascular disease. For instance, mean daytime sound pressure levels in excess of 60 db_A_ slightly increased the risk for ischemic heart diseases [44], while we have reported an association between long term exposure to primarily traffic-related noise and coronary heart disease mortality in Vancouver [45]. The low traffic environment and higher green space exposure of the Residential route may have impacted stress and anxiety levels [46], and measures of blood pressure while cycling this route [46]. A study conducted in London comparing the effects on respiratory function after walking along high vs. low traffic routes concluded that exposure to higher pollution levels negated any potential health benefits from walking [47]. Future research should explore how route characteristics such as these may play a role in affecting measured health variables. In addition, it would be valuable to understand how long the acute effects of these exposures may persist and to evaluate the potential for more longer-term impacts of repeatedly traveling on high versus low traffic routes on cyclists’ health. Such research may help inform both urban design more generally (e.g. a potential need to separate cyclist infrastructure and routes from sources of air pollution and noise) as well as the design and location of cycling routes specifically (e.g. a potential benefit of greenness).

The overall magnitude of exposures encountered along the two routes in our study (Downtown route mean = 16,870 pt/cm³; Residential route mean = 10,840 pt/cm³) were somewhat lower than measured on other studies of cyclist exposures, although the contrast between the routes was of similar relative magnitude. Jarjour et al. found no changes in lung function after comparing cycling along high (mean concentration = 19,945 pt/cm³) and low (13,517 pt/cm³) traffic routes in Berkeley, USA [48]. Strak et al. measured respiratory symptoms, exhaled nitric oxide and lung function changes after cycling high (44,090 pt/cm³) and low (27,813 pt/cm³) traffic routes in Utrecht, The Netherlands. PNC levels were associated with post-pre ride decreases in peak expiratory flow (PEF), but not with any of the other measures [26]. The overall absence of impacts on respiratory health endpoints was consistent with our findings, especially considering the overall higher exposure levels and larger differences between routes in the Dutch study. Similarly, Zuurbier et al., in a similar route comparison study (high traffic: 48,939 pt/cm³, low traffic: 39,576 pt/cm³) in Arnhem, The Netherlands reported an association between PNC exposure with decreased PEF and increased airway resistance, but no changes in other lung function parameters or exhaled nitric oxide [49]. In some cases these differences in particle counts may be due to different particle size limits of the instruments used to measure PNC; while we used the P-trak 8525 (range of 20 nm to 1 μm), other studies used models of mobile condensation particle counters with a broader range of 10 nm to > 1 μm [49].

Although we did not identify other cyclist studies with measures of endothelial function, in the study conducted in Arnhem, a weak positive association was observed between PNC and CRP levels but results for other biomarkers (IL-6/8/10, tumor necrosis factor-alpha, Clara cell protein 16, blood cell counts and blood coagulation markers) were null [50]. Weichenthal et al., reported associations between PNC exposures during cycling with FEF_25–75_ and exhaled nitric oxide, although the comparisons included a clean indoor (1162 pt/cm³) cycling trial in addition to cycling on high (19,747 pt/cm³) and low (10,882 pt/cm³) traffic routes in Ottawa, Canada [27]. This study also reported associations between PNC exposure with measures of heart rate variability, similar to a study in Dublin, Ireland where cyclist exposures were compared to those of other commuting modes [25].

This study included participants of a variety of fitness levels, which may have resulted in the inconsistent magnitude or direction of the blood biomarker results. There is evidence that stress-associated biomarkers may respond in dissimilar ways due to effect modification by fitness level [51, 52]. Study limitations include the use of a small sample size, which limits power to detect any differences. Because we did not measure the maximal aerobic capacity (V˙O_2_max) of the participants, we were unable to objectively quantify the fitness level of participants, which prevented us from adjusting for this variable. Furthermore, it is possible that only individuals who are frequent cyclists with interests in the topic of air pollution chose to participate in this study, limiting generalizability of the results to people who cycle less frequently or for leisure, or to cyclists outside the study age group of 19–39 years including those that may live with chronic health conditions or that take medications excluded by this study protocol. In addition, RHI is a surrogate measure of vascular function and while generally consistent with other predictive measures of cardiovascular risk [53], it is susceptible to effects of increased sympathetic activation due to environmental discomfort and not directly comparable to other measures of vascular function such as flow-mediated dilation [54, 55]. Further, RHI was measured immediately following exercise and we were therefore not able to assess how exposure impacts may have affected the various measures in the hours following the conclusion of our testing and it is uncertain how long the effects we observed with RHI would continue to persist following exposures and cycling activity.

## Conclusions

Given the individual benefit of improving physical fitness and the societal reward of reducing healthcare costs associated with improved fitness and air quality [8, 56], the estimated benefits due to cycling outweigh the associated risks [57–59]. From this perspective it is advisable to support cycling in the conditions described along either of these route types. Cycling either route provides the benefit of physical activity, and neither route was found to conclusively lead to adverse measures of the surrogate health endpoints that were measured in this study. With regard to heterogeneity between route types, there may be advantages to cycling along routes that require additional effort or that have lower air pollution levels. A better understanding of other potential health influencing factors while cycling is needed to inform public health messages to cyclists in selecting routes with the most beneficial features, as well as to inform planning and policy when designing cycling routes.

## Abbreviations

8-OHdG: 8-hydroxy-2′-deoxyguanosine
BMI: Body mass index
CRP: C-reactive protein
GM: Geometric mean
IL-6: Interleukin 6
PEF: Peak expiratory flow
PM: Particulate matter
PNC: Particle number concentration
RHI: Reactive Hyperemia Index
SD: Standard deviation
$$ {\dot{\mathrm{V}}}_{\mathrm{E}} $$: Minute ventilation
$\dot{\mathrm{V}}{\mathrm{O}}_2\max$: Maximal aerobic capacity
WHO: World Health Organization
